# Tuning in to Kids^®^ for parents of preschool-aged children in Portugal: study protocol for a randomized controlled trial

**DOI:** 10.1186/s40359-026-04450-4

**Published:** 2026-04-16

**Authors:** Stephanie Alves, Margarida Faria, Tiago Miguel Pinto, Ana Rita Cruz, Cláudia Sousa, Ana Beato, Sara Magalhães Albuquerque, Vitória Dias, Inês Jongenelen, Diogo Lamela, Raquel Costa, Raquel Pires

**Affiliations:** 1 HEI‐Lab: Digital Human-Environment Interaction Labs, Lusófona University, Lisbon, Portugal; 2 Piaget Institute, Insight - Piaget Research Center for Ecological Human Development, Lisbon, Portugal; 3 Faculty of Psychology and Education Sciences, Center for Research in Neuropsychology and Cognitive and Behavioural Intervention (CINEICC), University of Coimbra, Coimbra, Portugal

**Keywords:** Tuning in to Kids^®^, Parental emotion socialization, Randomized controlled trial, Preschool, Children's emotional and behavioral difficulties

## Abstract

**Background:**

Childhood mental health (MH) problems are a major public health concern due to their high prevalence and well-documented adverse consequences, and are often under-recognized and untreated. Less effective parental emotion socialization practices play a critical role in the emergence of childhood MH problems, influencing children’s socioemotional development. Emotion-focused parenting programs, such as Tuning in to Kids^®^ (TIK), could be a promising approach to promote parents’ emotion coaching skills, enabling children to understand and regulate their emotions more effectively, while also supporting parents in managing their own emotions. Prior evidence supports TIK’s efficacy in improving children’s and parents’ MH outcomes across several countries; however, TIK’s efficacy has not yet been explored in Southern Europe. This study aims to provide a process and efficacy evaluation of TIK for parents of preschool-aged children in Portugal.

**Methods:**

This trial is a parallel two-arm randomized clinical trial, which includes both process (i.e., feasibility and acceptability) and efficacy evaluation (i.e., change in primary and secondary outcomes and moderated effects) of Tuning in to Kids^®^ in Portugal. A minimum of 152 parents/legal guardians (> 18 years old) with at least one child between 3 and 6 years old will be enrolled. Parents will be randomly assigned to the intervention group or waiting list control group (76 per condition), through blocked randomization, with allocation concealment. Participants in both conditions will complete self-report questionnaires assessing primary (parental emotion socialization) and secondary (parents and children functioning dimensions) outcomes at baseline, post-intervention, and follow-up (2 months post-intervention). Longitudinal data will be analyzed using linear mixed-effects models accounting for repeated measures within participants.

**Discussion:**

We anticipate that the Portuguese version of Tuning in to Kids^®^ will be feasible, acceptable and effective in improving parent emotion socialization (e.g., more adjusted parents’ beliefs and reactions to children’s emotions and emotion regulation skills), and parents’ and children’s psychological functioning (e.g., less parenting stress and reduced children’s emotional/behavioral difficulties). If achieved, these findings will support TIK’s use in promoting MH among families.

**Trial registration:**

The clinical trial is registered at ClinicalTrials.gov (Identifier: NCT07350083, date assigned January 13, 2026).

**Supplementary Information:**

The online version contains supplementary material available at 10.1186/s40359-026-04450-4.

## Introduction

Mental health (MH) problems in early childhood represent a growing public health concern, affecting approximately 17.6% of preschool-aged children worldwide [1]. Internalizing and externalizing problems in young children not only lead to lower academic attainment and compromised social interactions with peers but also carry long-term consequences, including poorer quality of life, impaired MH, and lower life satisfaction in adulthood [2, 3]. Although early prevention of, and intervention in, MH problems are crucial to promote optimal child development [4], access to timely and appropriate support services remains limited, leaving a considerable proportion of children without access to appropriate care, particularly in low-income families [5].

Difficulties in emotion regulation represent a transdiagnostic symptom underlying a wide range of MH problems [6] and, therefore, constitute a critical target for intervention [7]. Early parent-child interactions, particularly how parents respond to their children’s emotions and model emotional regulation, play a critical role in shaping children’s emotional competence [8]. Accordingly, interventions that enhance supportive emotion socialization parenting practices (ESPP) offer a promising venue for preventing early MH problems.

Emotion-focused parenting programs, such as Tuning in to Kids^®^ (TIK; [9]), enable children to better understand and regulate their emotions by supporting parents in managing their own emotional responses and promoting the use of emotion coaching. TIK is an evidence-based program delivered by trained facilitators in six weekly, two-hour group sessions, originally designed for parents of preschoolers [9].

Central to the program is the concept of *meta-emotion*, which refers to parents’ beliefs and behaviors regarding emotions, shaped by their past experiences and family of origin. Meta-emotion influences how parents respond to their children’s emotions and behaviors, thereby informing ESPP toward children’s emotional expression. These practices can be either supportive (*emotion coaching*) or unsupportive (*emotion dismissing*) of the children’s emotional development. Unsupportive ESPP involve parents minimizing, criticizing, or avoiding children’s emotional experiences, thus hindering the development of emotional regulation. In contrast, supportive ESPP involve parents validating children’s feelings, capitalizing emotional situations as opportunities for learning, to coach emotional understanding and regulation. Such supportive parenting practices promote children’s emotional development, strengthen parent-child relationships, and are associated with positive outcomes for parents, including greater emotional awareness [10]. The program has been shown to improve child and parent MH outcomes across several countries [11, 12].

Despite its growing evidence, TIK feasibility, acceptability and efficacy have not yet been explored in Southern European countries, namely in Portugal. This is particularly relevant considering the high prevalence of childhood MH problems reported in some of these countries (e.g., Spain) and lack of access to public MH services, especially amongst families facing socioeconomic adversity [13]. In Portugal, mental healthcare systems are often under-resourced, with long waiting lists and limited availability of specialized child MH services (e.g., [14]). Portugal has been identified as one of the Southern European countries with the highest levels of unmet healthcare needs and the widest socioeconomic disparities in access to services [15]. Socioeconomic inequalities remain a critical concern, as financial hardship and restricted access to supportive interventions place many families at heightened risk for early mental health difficulties [16]. Additional barriers to access to MH care include an insufficient supply of clinical staff and a lack of standardized referral criteria, with waiting times for pediatric psychiatric consultations averaging nearly four months in Portugal [17]. Thus, emotion-focused parenting programs, such as TIK, when delivered in the community, may offer a cost-effective strategy to address the observed alarming MH care disparities.

Equally important are the cultural differences in emotion socialization, which reinforce the need to explore the feasibility, acceptability and efficacy of emotion-focused parenting interventions in different countries. Parenting practices vary considerably across regions, with meaningful implications for children’s emotional development (e.g. [18]), . In Nordic countries, parents are more likely to value autonomy over control and tend to adopt less authoritarian parenting [19]. Such supportive practices are associated with higher children’s self-esteem and more adaptive socioemotional functioning [20].

By contrast, in Southern European countries, like Portugal and Italy, parental overprotective approaches are more frequent, characterized by higher levels of control (e.g. [21, 22]), . Although collectivist and interdependent cultures may encourage the expression of socially engaged emotions, such as empathy and guilt [23], overprotective parenting practices may also restrict opportunities for independent growth and hinder children’s development of effective emotion self-regulation [24]. In addition, despite its legal prohibition [25], harsh disciplinary strategies such as physical punishment are highly prevalent amongst Portuguese parents [26]. Adapting parenting programs to account for these cultural differences may thus help to promote greater equity in access to MH care in Southern European Countries by making such interventions broadly accessible and culturally sensitive [27].

Ensuring access to effective parenting programs is particularly relevant for disadvantaged families, where parents often face additional challenges that compromise their MH and their ability to provide adequate care [28]. At the same time, children in these families are frequently exposed to early adversity [29], associated with increased behavioral problems, which can further elicit harsher and more inconsistent parenting practices [27] and, in turn, reinforce children’s emotional and behavioral difficulties [30].

Particularly, parents’ higher sociodemographic disadvantage (e.g., lower age, education, and income) predicts lower supportive emotion socialization, as the additional stressors and limited resources of these contexts may undermine parents’ ability to cope with parenting demands and provide supportive emotional responses [31]. As a result, MH outcomes are generally poorer among these families and largely overlooked [32], which limits children’s ability to reach their full potential as adults [33]. Particularly in Portugal, where nearly 379,000 children (22.6%) are at risk of poverty and social exclusion, highlighting an urgent need for child support [13].

Parenting interventions may also provide an effective strategy to address such disadvantages. Evidence-based parenting programs can improve parenting practices and overall family functioning, as well as reduce socioemotional problems in children from disadvantaged, high-risk families (e.g., [34]. However, engagement in parenting programs is particularly challenging for families with low socioeconomic status, with prior studies showing lower attendance rates [35]. Financial costs and lower education levels are associated with poor parental attendance [36]. As a result, parents with the greatest potential to benefit from parenting programs (e.g., [37]) are often the least likely to enroll [35].

Thus, efforts to develop evidence-based, accessible, and appealing parenting programs to low-SES families are critical. Rigorous outcome evaluations, including the use of control groups and follow-up assessments, are needed to establish the short- and long-term efficacy of such interventions [38]. By fostering greater engagement amongst disadvantaged families, emotion-focused parenting programs, such as TIK, may promote supportive ESPP to improve the family environment, parental MH, and children’s emotional and behavioral difficulties. Over time, these benefits may help to reduce inequalities in MH and address critical gaps in child MH provision in Portugal.

## Objectives

Framed within the Getting To Outcomes framework [39], this trial aims to conduct a process and efficacy evaluation of TIK among parents of preschool aged children in Portugal. Regarding process evaluation, we will evaluate TIK’s (a) feasibility (e.g., adherence to the program, whether TIK is implemented as intended and enablers and barriers to TIK implementation), and (b) acceptability (e.g., satisfaction, suggestions for improvement). Regarding efficacy, we will assess whether the TIK will be able to improve parental emotion socialization (primary outcomes, e.g., parents’ reactions to children’s emotions, parental emotion regulation) and parents’ and children’s emotional and behavioral functioning (secondary outcomes, e.g., parenting stress, children’s emotional/behavioral problems). A waiting list control group will be used. We hypothesize that parents receiving TIK will (1) show improvements in parental emotion socialization and (2) report improvements in parental and children functioning, across time (i.e., from pre-intervention to follow-up), compared with parents of the waiting list control group. We will also explore whether families’ psychosocial risk moderate the TIK’s efficacy on primary and secondary outcomes.

## Methods

### Trial design

The clinical trial is registered at ClinicalTrials.gov (Identifier: NCT07350083, date assigned January 13, 2026). Ethical approval to conduct this clinical trial was obtained from the Ethics Committee of the School of Psychology and Life Sciences of Lusófona University (Ref. CEDIC-2025-10-30/CEDIC-2025-5-35). The clinical trial will be conducted in compliance with the American Psychological Association [40] guidelines and the Declaration of Helsinki [41]. The study protocol of this clinical trial follows the SPIRIT (Standard Protocol Items: Recommendations for Intervention Trials) guidelines [42] and the reporting of the RCT will followCONSORT guidelines [43].

This study is a parallel two-arm randomized controlled trial that will be conducted between December 2025 and December 2027. After completing the baseline assessment, participants will be randomly allocated to either the intervention group or the control group (allocation ratio 1:1). A wait-list group will be used to minimize threats to internal validity and because it has been considered ethically acceptable for preventive interventions [44]. Participants in the control group will receive the intervention after the follow-up assessment.

### Trial setting

This trial will be conducted in the Lisbon Metropolitan Area or Lisbon District (Portugal). Findings from a previous study showed that parents expressed a preference for sessions held in familiar community settings, such as their children’s schools or local health centers, and after work [45]. Accordingly, protocols were formally established with several community partners, and TIK sessions will be held in community settings (e.g., schools, hospitals) to facilitate participants’ enrollment and engagement.

### Participants

Eligible participants will be parents/legal guardians (hereafter referred to as “parents”) (1) with at least one child aged between 3 and 6 years old (inclusive) who lives with the parent, (2) at least 18 years old, (3) who can read, understand, and speak Portuguese, and (4) who live in the Lisbon Metropolitan Area or Lisbon District. When requested, both parents will be allowed to participate. Exclusion criteria include (1) parents’ being currently enrolled in another structured parenting program, (2) the child is already attending elementary school at the time of recruitment, (3) the child has a diagnosis of a severe Communication and/or Developmental Disorder, or (4) the child is under a child protection measure that implies that the child does not live with their parents (e.g., if the child is in foster care). Parents who have multiple children within the target age range will be instructed to respond with reference to the child for whom they experienced the most concern.

### Study procedures

The clinical trial procedures (enrollment, intervention and assessments) are detailed in Table 1. Eligible parents who agree to participate in the project (informed consent) will complete baseline in-person questionnaires in the place where the sessions will be held. After that, they will be randomly assigned to either the intervention program condition (TIK) or the control condition. Participants in the intervention group will start TIK sessions within 1 month following the baseline assessment and will complete post-intervention and follow-up (2-month post-intervention) assessments in person. At 2-month post-intervention, a booster session will be held at the same location the sessions took place immediately after participants complete the protocol evaluation. According to the original TIK structure, this booster session is offered as an optional follow-up to consolidate skills [10]. In the present clinical trial, we chose to include this session not only to minimize parents’ attrition but also to provide participants with an additional opportunity to practice skills and discuss challenges beyond completing questionnaires. Participants in the control group will complete the same evaluation protocol at equivalent assessment time points, in the same setting as the intervention sessions, but on different days to minimize contamination between study arms. In the case of both parents participating, each will complete an evaluation protocol independently. The facilitators will complete questionnaires during the intervention (after each session) and at the end of each facilitated group assessing implementation fidelity.

**Table 1:** Clinical trial procedure

Variables	Informant	Measures	Enrollment and screening	Baseline [T0]	Allocation	During intervention	Post-intervention [T1]	2-month Follow-up [T2]
Eligibility screening	Parent	-	*					
Informed consent	Parent	-	*					
Sociodemographic and clinical data (parent and child)	Parent	Questionnaire developed for this study		*				
	Facilitators	Questionnaire developed for this study		*				
Process evaluation								
Fidelity	Facilitators/independent observer	TIK fidelity checklist				*		
Feasibility	Parent	Questionnaire developed for this study					*	
Attendance and retention of participants in the intervention	Facilitators	Facilitators’ records				*		
Acceptability	Parent	Questionnaire developed for this study					*	
Outcome evaluation								
Primary outcomes								
Parental emotion socialization								
Parents’ reactions to children’ emotions	Parent	CCNES		*			*	*
Parents’ beliefs about children’ emotions	Parent	PBACE		*			*	*
Parental emotion regulation	Parent	IM-P		*			*	*
Self-regulation	Parent	DERS-SF		*			*	*
Secondary outcomes								
Child								
Emotional/behavioral problems	Parent	SDQ-P		*			*	*
Emotional regulation	Parent	ERC		*			*	*
Parent/Legal guardian								
Parenting stress	Parent	PSS		*			*	*
Depressive symptoms	Parent	HADS - D		*			*	*
Parenting self-efficacy	Parent	MaaP		*			*	*

‘Parent’ refers to parents and legal guardians

*CCNES* Coping with Children’s Negative Emotions Scale, *PBACE* Parents’ Beliefs About Children’s Emotions Questionnaire, *SDQ-P* Strengths and Difficulties Questionnaire – Parent, *ERC* Emotion Regulation Checklist, *DERS-SF* Difficulties in Emotion Regulation Scale - Short Form, *PSS* Parental Stress Scale, *HADS-D* Hospital Anxiety and Depression Scale - Depression Symptoms, *MaaP* Me as a Parent Questionnaire, *IM-P* Interpersonal Mindfulness in Parenting Scale

### Participants enrollment and screening

Participants will be recruited through self-referral and referral processes from collaborating community partners (e.g., child welfare agencies, family support and counselling services, schools, local health center units) from the Lisbon Metropolitan Area or Lisbon District. Collaborating institutions will be requested to share the project with all parents via newsletter, personalized emails and putting up advertisement flyers in strategic locations. The dissemination materials will include a link available through REDCap (Research Electronic Data Capture) - a secure electronic data capture system designed to support data entry and management for research studies - through which parents can manifest their interest in being enrolled in the project. Interested parents could also contact the research team by email or telephone. Professionals working with families, namely those working with more vulnerable families (e.g., psychologists, social workers), will also have the opportunity to refer eligible parents to the clinical trial, based on their judgment that the family could benefit from participating in an emotion-focused group intervention. If they agree and consent, their contact details will be shared with the research team. Broader dissemination of the project will also take place through collaborating community partners’ social media (e.g., Instagram, Facebook), which will contain a link to be filled out by interested parents. A research team member will further contact all interested parents by telephone to assess their eligibility, discuss availability and logistical preferences (e.g., session schedule, site, and childcare needs), inform them of their eligibility status, and provide a full explanation of the clinical trial procedures (e.g., study aims, assessments, confidentiality and ethics issues, participants’ rights). Particularly, all participants will be informed that they will be randomly assigned to one of two groups, meaning that they could initiate the intervention within approximately one month or after 4–5 months (after the follow-up assessment), and that their participation involves no costs. Immediately after the telephone interview, written information about the clinical trial will be sent to parents by email, and if they agree to participate, parents will be asked to return their written informed consent via the same way. Following the provision of informed consent, parents will complete the baseline assessment protocol. Non-eligible parents will be informed about the reasons for their exclusion during the telephone interview, and those who require professional support will be advised or referred to specialized care by the research team. The expected completion date of recruitment is 28th February 2027.

### Sample size

Power analyses were conducted using Monte Carlo simulations for linear mixed-effects models in R (version 4.4.0) (packages *lme4* and *simr*). For each simulation, medium-sized interaction effects were specified, and different random intercept and slope variances were considered. Power for fixed interaction effects was evaluated using Wald *t*-tests; power to detect inter-individual variability in change over time was evaluated by comparing the models that include a random slope for time to reduced models including only a random intercept. These comparisons were performed using likelihood ratio tests. For primary analyses examining the time x group interaction effect on TIK’s efficacy, the simulation results indicated that, to achieve 80% power at *α* = 0.05, a total sample size of approximately 132 participants is required. Although the study is powered primarily to detect group × time effects and to account for significant inter-individual variability in change over time, exploratory moderation analyses to examine whether pre-test psychosocial risk is a moderator of TIK’s efficacy are also planned. With 132 participants, the power to detect a three-way group x time x psychosocial risk interaction is substantially lower, as expected (approximately 49%), and approximately 286 participants are required to achieve 80% power. Since we anticipate difficulties in recruiting such a large sample, in this trial, findings concerning the moderator role of psychosocial risk will therefore be interpreted cautiously and considered exploratory, while still providing valuable preliminary evidence regarding which families may benefit most from TIK. These results will help generate hypotheses and inform the design of future, larger implementation trials. Considering an attrition rate of 15% [46, 47], we will have to recruit approximately 152 participants to complete the baseline assessment.

### Randomization and blinding

To ensure balanced groups throughout the enrollment period, randomization will be conducted using a permuted block procedure, with block sizes varying between 4 and 6, depending on the number of eligible parents. To ensure allocation concealment, the sequence will be generated through computer randomization software by an independent researcher not involved in participants’ enrollment or assessments. Group assignments will be further communicated to the research team, who will subsequently inform parents of their allocation via telephone. Additionally, groups’ equivalence will also be assessed regarding primary outcomes and key covariates. Considering the nature of the intervention and similarly to research evaluating TIK in other countries (e.g., [48]), this study will be an open-label trial, as neither the researchers nor the parents will be blinded to the participants’ group allocation.

### Patient and public involvement

Community partners will be involved in the design, conduct, and reporting of the trial. At a developmental stage, stakeholders will provide advice about effective recruitment strategies and dissemination plans, as well as anticipated barriers to recruitment and efforts to overcome them, to maximize successful participants’ engagement. During the implementation phase, stakeholders will lead the recruitment of participants through general dissemination channels and personalized referrals, and periodic meetings will be held to monitor the impact of the recruitment strategies. Community partners will also provide facilities for group sessions. The trial’s results, namely those related to process evaluation, will be discussed with the stakeholders to determine whether changes (and which) to TIK should be made to optimize its integration into routine, integrated care. At the midpoint of the project, one seminar directed to community partners will be held to monitor implementation of the project and make appropriate adjustments, if necessary, as well as to provide preliminary results. Tools for disseminating the trials’ results among the general public will also be co-created with community partners.

### The intervention

#### Tuning in to Kids^®^ Intervention

Tuning in to Kids^®^ is an evidence-based parenting program for children aged 3 to 12, designed to foster emotionally responsive parenting, thereby promoting children’s emotional development. Drawing upon research in the field of emotion socialization (e.g., [49, 50], the program is based on the premise that children’s MH difficulties can be reduced through the experience of emotional validation by parents. The TIK program is delivered as a structured, group-based intervention consisting of six 2-hour weekly in-person sessions [9]. A follow-up booster session is included in the program to allow parents to consolidate their learning and address current main challenges. The sessions are conducted by two facilitators in closed groups of 8 to 12 parents. After session 2, no additional participants were allowed to join the group, to facilitate peer support and open discussion. Program content combines psychoeducation, group brainstorming, role-play exercises, video materials, and home practice activities for parents to apply emotion coaching in real-life scenarios. The structured format (see Table 2) provides a supportive environment for parents to reflect on their own meta-emotion and progressively develop the skills to respond to their child ren’s emotions through emotion coaching [51]. More information about the program can be found at the website: https://tuningintokids.org.au/. The Portuguese version of TIK was adapted primarily to ensure linguistic and cultural relevance (i.e., after being translated into Portuguese, the materials were reviewed for cultural appropriateness). Only minor modifications were made, including the adaptation of some examples and references in the program content to better reflect Portuguese family contexts.

**Table 2:** Session-by-session description of the Tuning in to Kids^®^ intervention

Session	Themes
1	Introducing the program and the concept of emotion coaching, while creating a warm, non-judgmental environment that normalizes parental challenges and worries, builds emotional awareness, and welcomes participants.
2	Exploring emotion coaching and the concept of meta-emotion by helping participants to reflect on how past experiences with the family of origin and cultural values influence parenting practices and the use of empathetic responses.
3	Supporting parents to understand children’s emotional experiences around sadness by responding with empathy and validating feelings to promote emotional regulation.
4	Consolidating skills in self-care and responding to children’s fears during periods of heightened emotional activation.
5	Reflecting on children’s anger and parents’ own emotional self-regulation, to use emotion coaching to respond to challenging behaviors.
6	Consolidating learning by reviewing the program, reinforcing the five steps of emotion coaching and encouraging ongoing practice, self-care, and further growth.

Adapted from [52]

The group’s location, dates, and starting times will be determined according to participants’ preferences and availability and will be communicated to participants at least three weeks before intervention starts, followed by a reminder text message one day before the intervention begins. Participants will be able to discontinue study participation (1) upon request or (2) if the facilitators noticed some harmful effects (e.g., severe emotional activation). In such cases, participants will be asked if they want to receive another type of professional support and/or resources. Concomitant individual care for either parents or children is permitted during the trial. No new participants are admitted after the second session to ensure a safe and consistent group environment. Moreover, intervention groups will consist exclusively of parents enrolled in the trial.

To promote participant retention, when participants miss a session, facilitators will contact them by telephone to inquire about the underlying reasons for missed sessions and explore obstacles that may have hindered attendance. Wherever possible, the research team will provide solutions to overcome potential barriers and enhance participation (e.g., normalizing potential anxiety felt in a group intervention, providing childcare for siblings). Parents allocated to the waitlist control group will be regularly contacted throughout the waiting period by the research assistants, by telephone and SMS, to share updates on trial progress, and provide reminders of scheduled assessments and that they will receive the intervention after the follow-up assessment.

#### Training, supervision, and implementation fidelity

All facilitators will have at least a master’s degree in psychology and have completed the mandatory TIK professional training, being certified as TIK facilitators. The training program is designed to enhance knowledge and develop the essential skills for effective TIK delivery, thereby improving the qualification of TIK facilitators. Each facilitator will follow a standardized manual outlining specific procedures and activities for each session. Facilitators will receive supervision throughout delivery from the program authors (before session 1 and after sessions 2, 4, and 6) to enhance fidelity to the TIK program and give advice to overcome potential challenges (e.g., adherence to the program, time management). Facilitators will also have access to the Facilitator Portal (access restricted to certified TIK facilitators), which includes several supplementary tools to improve the program’s delivery. To evaluate whether TIK is delivered as intended and facilitate comparisons across groups, both facilitators and an observer element (who will be present in all groups delivered during the trial) will complete the TIK Program Fidelity Checklist at the end of each session. For each planned topic and activity, they will indicate if it was covered or not, with space provided for additional comments. Facilitators will make an effort to deliver any content missed in a given session in the session that follows. Facilitators will not be blinded to the participants’ condition.

## Measures

### Sociodemographic measures

A questionnaire specifically developed for this study will collect sociodemographic and health-related data at baseline, referring to the parents (i.e., sex, education, nationality, ethnicity, professional status, marital status, if currently in a marital relationship with the other child’s parent, monthly family income, receipt of financial support from public entities or others, perceived sufficiency of household income, receipt of support from social services, household size, history of MH problems) and the child (i.e., sex, age, and existence of mental, physical and/or developmental problem).

### Psychosocial risk

Psychosocial risk data will be collected through the sociodemographic questionnaire described above. A composite score will be computed by summing up the following risk conditions: (1) parental low education (≤ 9 years); (2) ethnic minority (non-White), (3) parental unemployment (currently or in the previous 12 months); (4) single-parent family, (5) low monthly family income (< 2 minimum national salaries); (6) parental receipt of financial support from public entities or others (yes v. no); (7) parental perception of insufficient household income to meet daily family needs (yes v. no), (8) parental receipt of support from social services (e.g., child protection services; yes v. no); (9) high household size (≥ 75th percentile of the sample distribution); (10) parental previous diagnosis of MH problems (yes v. no); and (11) child previous diagnosis of physical or developmental problems (yes v. no). The presence of each risk condition will be coded as 1 and absence will be coded as 0. Scores can range between 0 and 11 and higher scores reflect higher psychosocial risk.

### Outcome measures

Outcome measures will be assessed with the Portuguese Versions (PV) of the following instruments at specific time points (see Table 1).

### Primary outcome measures

#### Parental emotion socialization

##### Coping with Children’s Negative Emotions Scale (CCNES)

A modified version of the CCNES ([53]; PV: [54, 55]) will be used to assess parents’ emotion socialization behaviors. The scale consists of 12 hypothetical scenarios in which the child experiences a negative emotion (e.g., anger, sadness). For each scenario, parents rate their likelihood of responding in each of seven ways on a 7-point Likert scale, ranging from 1 (*very unlikely*) to 7 (*very likely*). The measure includes seven subscales: Emotion-Focused, Problem-Focused, Minimizing, Punitive, Expressive Encouragement, Distress, and Ignoring reactions. In the present study, items from the Distress subscale were reworded to enhance clarity, and the Ignoring subscale was included, following [56]. Items from the Minimizing, Punitive, Distress, and Ignoring subscales are summed to assess emotion dismissing responses (i.e., negative reactions), and items from the Emotion-Focused, Problem-Focused, and Expressive Encouragement subscales are summed to measure emotion coaching (i.e., positive reactions). A total score can be obtained for each scale by averaging the items (range 1–7). Higher scores in each of these categories indicate greater endorsement of that practice. In the Portuguese version, Cronbach’s alpha ranged from 0.83 to 0.89 [55].

##### Parents’ Beliefs About Children’s Emotions Questionnaire (PBACE)

The PBACE ([57]; PV: [58]) is a self-report measure designed to assess parents’ beliefs about children’s emotions (e.g., “*Showing anger is not a good idea for children*”). The questionnaire consists of 33 items rated on a 6-point Likert-type scale, with responses ranging from 1 (*strongly disagree*) to 6 (*strongly agree*). In the present study, four subscales will be used: Value of Anger (6 items; belief that anger can be valuable), Manipulation (4 items; belief that children’s emotional expression can be used to manipulate others), Control (5 items; belief that children can regulate their emotions), and Autonomy (7 items; belief that children should manage their emotions independently). Total scores will be computed for each of these subscales by averaging the respective items (range 1–6), with higher scores reflecting stronger endorsement of the corresponding belief in that category. In the Portuguese version, Cronbach’s alpha was 0.90 [58].

##### Interpersonal Mindfulness in Parenting scale (IM-P)

The subscale Self-regulation in Parenting of the IM-P ([59]; PV: [60]) will be used to assess parents’ ability to regulate their own emotions and behaviors in the parenting context. It is composed of 8 items (e.g. “*When I am upset with my child*,* I notice how I am feeling before I take action*”) rated on a 5-point Likert-type scale from 1 (*never true*) to 5 (*always true*). A total score will be obtained by summing up the items (range 8–40; items 1, 4, 5 and 8 will be first reverse-scored). In the Portuguese version, Cronbach’s alpha for this subscale was 0.83 [60].

##### Difficulties in Emotion Regulation Scale - Short Form (DERS-SF)

The DERS-SF ([61]; PV: [62]) is an 18-item self-report measure of difficulties in emotion regulation (e.g., “*I am confused about how I feel*”). Items are rated on a 5-point Likert-type scale ranging from 1 (*almost never*) to 5 (*almost always*). A total score (range 1–5) is obtained by averaging all items, with higher scores indicating greater difficulties. In line with the recommendations of the Portuguese authors [62], items 1, 4 and 6 will be excluded from the analyses. In the Portuguese version, Cronbach’s alpha was 0.93.

### Secondary outcome measures

#### Children

##### Strengths and Difficulties Questionnaire - Parent (SDQ-P)

The SDQ-P ([63]; PV: [64]) is a brief screening instrument designed to assess children’s emotional and behavioral difficulties. The parent-report version consists of 25 items (e.g., “*kind to younger children*”), each rated on a 3-point Likert-type scale, from 0 (*not true*) to 2 (*certainly true*), based on the child’s behavior over the past 2 months. Items 7, 11, 14, 21 and 25 are reverse-scored. The *Total difficulties* score (range 0–40) is calculated by summing the following subscales: Internalizing Problems and Externalizing Problems. Higher scores indicate greater difficulties. The Prosocial Behaviors subscale (range 0–10) is scored separately, as a measure of strength. Two versions of the scale (2–4 years old and 4–17 years old) will be presented to parents as a function of the child’s age. The scales differ slightly on items 18, 21 and 22 to ensure age-appropriate language. In the Portuguese version, internal consistency was acceptable for all subscales: Internalizing Problems: *α* = 0.72, Externalizing Problems: *α* = 0.79, Prosocial Behaviors: *α* = 0.70 [65].

#### Emotion Regulation Checklist (ERC)

The ERC ([66]; PV: [67, 68]) is a self-report measure to assess children’s emotion regulation competencies. The revised PV of the scale [68] consists of 20 items rated on a 4-point Likert-type scale, ranging from 1 (*never*) to 4 *(almost always*). The instrument includes two subscales: the Emotion Regulation (7 items), which assesses children’s emotional expression, empathy and self-awareness (e.g., “*is easily frustrated*”) and the Emotion Lability/Negativity (13 items), which measures children’s reactivity to negative emotions and emotional intensity (e.g., “*exhibits wide mood swings*”). Items 5, 9, 16 and 18 are reverse scored. For each subscale, a total score is obtained by the sum of the corresponding items (range 7–28 and 13–52, respectively). Higher scores on the Emotion Regulation scale reflect better emotion regulation, whereas higher scores on the Lability/Negativity scale indicate greater difficulties in emotion regulation. In the Portuguese version, Cronbach’s alphas ranged between 0.66 and 0.80 [68].

#### Parents

##### Parental Stress Scale (PSS)

The PSS ([69]; PV: [70]) is a self-report scale comprising 18 items to assess stress experienced by parents in relation to raising their children (e.g., “*I am happy in my role as a parent*”). Following the recommendations from the Portuguese version (Algarvio et al., 2018), items 1–2 and 17–18 will be excluded from the analyses. Responses are provided on a 5-point Likert-type scale from 1 (*strongly disagree*) to 5 (*strongly agree*). A total score (range 14–70) is calculated by summing all items, with items 5–8 being reverse-scored. Higher scores reflect greater levels of parenting stress. In the Portuguese version, Cronbach’s alpha was 0.76 [71].

##### Hospital Anxiety and Depression Scale (HADS)

The Depressive Symptoms subscale of the HADS ([72]; PV: [73]) will be used to measure parental depressive symptoms experienced over the past week. This self-report subscale consists of 7 items (e.g., “*I feel cheerful*”) rated on a 4-point Likert-type scale, with scores ranging from 0 to 3 (e.g., “*not at all*”, “*most of the time*”). The total score (range 0–21) is obtained by summing up the items, with higher scores indicating greater levels of depressive symptoms. In the Portuguese version, Cronbach’s alpha was 0.89 [74].

##### Me as a Parent Questionnaire (MaaP)

The Self-Efficacy subscale of the MaaP ([75]; PV: [76]) will be used to assess parents’ self-efficacy in parenting. The subscale has 4 items (e.g., “*My parenting skills are effective*”), rated on a 5-point Likert-type scale, from 1 (*strongly disagree*) to 5 (*strongly agree*). A total score is obtained by summing up the items (range 4–20), with higher scores reflecting higher perceptions of parenting self-efficacy. In the Portuguese version, the Cronbach’s alpha for this subscale was 0.78 [77].

### Other outcome measures

#### Feasibility of the intervention

TIK’s feasibility will be evaluated in terms of implementation fidelity, adherence to the intervention and facilitators and barriers to TIK implementation. Adherence will be estimated through the number of sessions attended; those who attend at least four of the six sessions will be considered completers (67%), the remaining will be considered non-completers. Participants will be asked to answer two questions on facilitators and barriers to TIK implementation at post-intervention (*What factors made it easier for you to participate in the program? What factors made it more difficult for you to participate in the program?*). When applicable, the time of the dropout from the intervention will also be recorded (e.g., after session 4, 5, 6 or at follow-up).

#### Acceptability of the intervention

TIK’s acceptability will be evaluated at the end of the program. Participants will be asked to complete a questionnaire composed of 16 questions answered on a Likert-type scale ranging from 1 (*not at all*) to 5 (*very much*), covering: (a) overall satisfaction with TIK; (b) if TIK meets parents’ expectations and contributes to improvements in their confidence as parent, in their relationship with their children, in their children well-being, and in their capacity to understand their children’s emotions and how to manage them; (c) the frequency to which they apply TIK contents on their daily interactions with their children; (d) the adequacy of the number, frequency and length of the sessions; (e) satisfaction with the group format; (f) perception of TIK’s facilitators competence and intervention’s facilities; and (g) usefulness of acquired strategies for parenting in the long-term. Participants will also be asked to report if they would recommend the program to other parents on a yes/no scale, as well as to rate the degree of facility/difficulty in understanding the concepts of emotion coaching and in applying emotion coaching strategies on a daily basis, using a Likert-type scale ranging from 1 (*very easy*) to 5 (*very difficult*). They will also be asked to answer three open-ended questions to report on the most and least useful aspects of the program, and if there were other contents that they would like to address in the program. A last open-ended question inquires about other suggestions for improving TIK.

### Harms

TIK has been investigated and applied worldwide and has been translated into 15 languages [10], and no risks or adverse effects have been documented to date (e.g. [12, 51]), . The facilitators will monitor participants’ well-being across the group sessions through open-ended questions made individually (separately from the group) after sessions 2, 4, and 6. Participants who missed one session will receive a phone call from the facilitators to monitor reasons for the missed session and inquire about well-being.

### Data management

Data management of this clinical trial will follow Lusófona University’s data protection policies and the General Data Protection Regulation (GDPR - UE, 2016/679). Research assistants will be responsible for data collection and data entry. Data from paper questionnaires will be entered on REDCap. By including range checks, field validation, mandatory fields, logic rules, and indicators of missing values, among other features, REDCap allows enhancing data quality in terms of its accuracy, completeness, and reliability. To maximize data accuracy, two independent research assistants will enter the same paper questionnaires into REDCap, which will compare the two entries and signal any discrepancy. Paper questionnaires will be kept at Lusófona University (access restricted), and any identifying data will be removed. Each participant will receive an ID code that would allow pairing data across assessment points. Pseudonymized data will be stored at Lusófona University server, after being encrypted and accessed by password, as well as signed consent forms. Only research assistants and project coordinators will have access to the data. Participants will have the right to access, change, or delete their own data at any time, as well as to remove their consent for participation, with no need for explanations. Data will be maintained for the time strictly necessary for achieving this project’s aims.

### Statistical methods

#### Process evaluation

Descriptive statistics (mean ± standard deviation and frequencies) will be computed for each item of the acceptability questionnaire using the Statistical Package for the Social Sciences (SPSS, IBM Corp., v.30.0). Responses to the open-ended questions of this questionnaire, as well as those referring to facilitators and barriers to TIK implementation, will be examined through thematic analysis following a deductive–inductive approach informed by the stepwise framework proposed by Braun and Clarke [78].

#### Efficacy evaluation

The statistical analyses will be carried out using SPSS and the R software (version 4.4.0). The level of statistical significance will be set at *p* < .05. Descriptive statistics will be computed for all study variables. Pearson/Spearman correlations will be conducted to explore associations between all the study variables at each assessment wave. Pre-test differences between the intervention and control groups will be analyzed regarding sociodemographic and health characteristics (using qui-square tests) and the primary and secondary outcomes using t-tests (for dichotomous independent variables) and one-way ANOVAs (for categorical and ordinal independent variables) to identify potential covariates.

Linear mixed-effects models accounting for repeated measures within participants will be tested to analyze TIK’s efficacy. Time 0 will be defined as the date of the pre-test assessment, time 1 will be the date of the post-test, and time 2 will be the date of the follow-up assessment. Time, group conditions (intervention (coded as 1) vs. control (coded as -1)), and the 2-way interaction time x group conditions will be included in the models as fixed effects. Moreover, for children-related outcomes (i.e., children’s emotional and behavioral functioning), the child will be treated as the primary unit of analysis and models will also include a random intercept and a random slope for time at the child level in order to account for between-child variability in baseline (at pre-test) levels of the outcome variables and between-child variability in change across time. Since both parents may participate in the study, the child-level random effects also allow one to take into account the non-independence of both parents’ reports for the same child. For parents-related outcomes (i.e., parental emotion socialization and parents’ emotional and behavioral functioning), the parent will be treated as the primary unit of analysis and models will include a random intercept and a random slope for time at the parent-level to account for between-parent variability. To account for the nesting of both parents within the same family, a random intercept at the child level will be included.

Exploratory moderation analyses will be performed in order to examine whether pre-test psychosocial risk is a moderator of TIK’s efficacy. Psychosocial risk, the 2-way interaction time x psychosocial risk, the 2-way interaction group conditions x psychosocial risk, and the 3-way interaction time x group conditions x psychosocial risk will be added to the previous linear mixed-effects models as fixed effects. Significant interactions with the continuous predictor (i.e., psychosocial risk) will be interpreted and graphed using one standard deviation above and below the grand mean as high and low values.

All linear mixed-effects models will be estimated using maximum likelihood, and the models’ assumptions will be validated by analyzing residual graphs. Deviance difference tests will be performed between a baseline growth model with only time as a fixed effect and models with the predictors to examine model fit improvements. All models will be adjusted for the covariates identified in the preliminary analyses. One model per outcome variable will be performed. Effect size measures will be calculated, namely the ICC (intraclass correlation coefficient) for random effects and Cohen’s *f*^2^ for fixed effects, following the guidelines of Lorah [79]. These analyses, including the calculation of the effect sizes, will be performed using the package *lme4* from R.

### Nonadherence, attrition and missing data

Participants who discontinue attending TIK’s sessions will be invited to complete all outcome assessments, in-person, in a local of their preference. Participant’s attrition from the trial will be estimated through the number of participants who do not complete post-intervention and/or follow-up assessments. All participants who completed the baseline assessment will be considered in the analyses, irrespective of their adherence to the intervention, following intention-to-treat (ITT) principles to minimize attrition bias. Secondary sensitive analyses will be conducted to assess differences between intervention completers and non-completers participants in terms of sociodemographic and health characteristics. Moreover, if substantial non-adherence to the intervention is observed, a secondary and exploratory complier average causal effect (CACE) analysis will be undertaken to estimate the intervention effect among compliers (i.e., participants who attended at least 4 of the 6 sessions, including three of the four core sessions - sessions 2, 3, 4 or 5 - which is considered the minimum exposure to receive TIK core components).

Researchers will carefully check the questionnaires at the time of completion and alert participants whenever missing responses are identified. Nevertheless, if missing data are present in the predictor variables of the linear mixed-effects models, the pattern of missingness will be analyzed, and when MAR (Missing at Random) or MCAR (Missing Completely at Random), multiple imputation will be performed using *mice* package from R prior to fitting the linear mixed-effects models. Missing data in the outcomes are handled by the *lme4* package, since it uses all available data through its Maximum Likelihood estimation.

### Data and trial monitoring

Data and trial monitoring will be led by the project’s coordinator. In order to continuously improve the implementation of the trial, the following activities are planned: realization of periodic meetings of the research team to monitor compliance with scheduled activities, discuss challenges and adjust action plans (one per trimester); preparation of interim progress reports with partial results, including satisfaction of families/facilitators and identification of risks/challenges; and realization of restricted and/or extended meetings with community partners in order to return partial results and planning of future actions. Throughout the trial, the facilitators and research assistants will pay close attention to any signs of discomfort among participants, and the program’s sessions and data collection procedures will only continue if participants are comfortable. Participants will be encouraged to share with the facilitators and research team, during the session or via individual contact, any discomfort they might feel during the trial. If necessary, the research team will provide guidance regarding alternative professional help.

### Ethics and dissemination

The clinical trial will be conducted in accordance with the GDPR of the European Union and in compliance with the American Psychological Association [40] guidelines and the Declaration of Helsinki [41]. The TIK protocol, including manifestation-of-interest forms, informed consent, questionnaires, and interview guides were submitted for review and approved by the Ethics Committee of the School of Psychology and Life Sciences of Lusófona University (CEDIC‑2025‑10‑30). Protocol modifications (e.g., changes in eligibility criteria or data collection methods) were documented in an amendment and (re)submitted for approval to the Ethics Committee of the School of Psychology and Life Sciences of Lusófona University prior to implementation. All amendments were approved (CEDIC-2025-5-35). Any amendments that may influence participants’ willingness to continue (e.g., additional assessments) will be explicitly explained, and renewed consent will be sought when required. Moreover, if a new amendment would be required, the same procedure will be conducted (i.e., submission for approval by the Ethics Committee of the School of Psychology and Life Sciences of Lusófona University prior to implementation).

Informed consent will be obtained from all participating parents or other legal caregivers/guardians prior to the beginning of any study-specific procedures. Potential participants will complete a manifestation-of-interest form, after which a trained member of the research team will contact them by telephone to confirm eligibility and provide a detailed verbal explanation of the study, including its objectives, procedures, randomization to intervention vs. wait‑list, assessment time-points, potential risks and benefits, confidentiality, and the voluntary nature of participation. The written informed consent form will then be sent by email; participants will be given adequate time to read the document and may seek clarification by email, telephone or during a brief online or in-person meeting and will be enrolled in the study only after the signed informed consent form is returned to the research team. Participation is voluntary and free of charge, and participants may withdraw at any time without any consequences.

The trial does not entail the collection of biological specimens. Personal information will be handled in accordance with the General Data Protection Regulation of the European Union and with institutional data protection policies. Contact details and signed informed consent forms will be stored in a password‑protected folder in the Universidade Lusófona cloud environment, accessible only to the principal investigator and the two research fellows responsible for participants’ recruitment and data collection; these files will constitute the unique link between participant identity and a randomly generated participation code and will be destroyed after the conclusion of data collection. All questionnaire datasets and other research data will contain only the participation code (i.e., will be pseudonymized) and will be stored in a separate password‑protected folder, whereas paper questionnaires will not include direct identifiers and will be kept in a locked cabinet at the HEI‑Lab, accessible exclusively to the principal investigator and the two research fellows. Data will be retained only for the period strictly necessary for the purposes of the project and will subsequently be irreversibly anonymized, in accordance with the requirements of the Ethics Committee of the School of Psychology and Life Sciences of Lusófona University and the project funder.

The intervention is preventive in nature and is considered to involve minimal risk; however, the discussion of emotions and parenting practices may occasionally elicit emotional discomfort. Participation in group sessions will be facilitated by clinical psychologists trained in the Tuning in to Kids^®^, who will actively monitor participants for signs of distress and provide immediate support, when necessary; if indicated, participants will be informed about and, upon request, referred to, appropriate specialized mental health services; moreover contact information for the research team will be made available for additional support. Participants allocated to the control (wait‑list) group will be offered the TIK program upon completion of the final follow‑up assessment, thereby ensuring equitable access to the potential benefits of the intervention. Any adverse events or unintended effects will be systematically documented and addressed according to institutional clinical and ethical procedures.

The results of this clinical trial will be communicated through different formats to address the needs of different audiences. Participants will receive a summary of the main findings (including whether the program showed benefits for parents and children). Social services and education professionals, as well as community partners (e.g., family support centers), will be provided with reports summarizing key results and implications for professional practice. Scientific findings will be disseminated via peer‑reviewed journals, master’s theses, presentations at national and international conferences, and events targeting the general population. No individual-level data will be disclosed to referring institutions, and participants’ identities will be anonymized in all dissemination materials.

## Conclusion

Findings from this clinical trial are expected to inform about the suitability and relevance of a widely used evidence-based emotion-focused group program for parents in Portugal, offering a cost-effective way to reduce the need for individual treatments (including psychiatric hospitalizations and medication costs) and alleviate the burden on already strained public MH services. Also, they might promote greater awareness and availability of parenting support, particularly relevant for under-resourced or underserved communities, thus helping to promote equity in access to MH care for children and families.

## Supplementary Information

Supplementary Material 1.

## Data Availability

No datasets were generated or analysed during the current study.

## Abbreviations

CCNES: Coping with Children’s Emotion Scale
DERS-SF: Difficulties Emotion Regulation Scale Short Form
ERC: Emotion Regulation Checklist
ESPP: Emotion Socialization Parenting Practices
GDPR: General Data Protection Regulation
HADS: Hospital Anxiety and Depression Scale
IM-P: Interpersonal Mindfulness in Parenting Scale
MaaP: Me as a Parent Questionnaire
MAR: Missing at Random
MCAR: Missing Completely at Random
MH: Mental Health
PBACE: Parent´s Beliefs about Children´s Emotions Questionnaire
PSS: Parental Stress Scale
PV: Portuguese Version
RCT: Randomized clinical trial
SDQ-P: Strengths and Difficulties Questionnaire-Parent
TIK: Tuning in to Kids^®^

## References

[1] Charach A, Mohammadzadeh F, Belanger SA, Easson A, Lipman EL, McLennan JD, et al. Identification of preschool children with mental health problems in primary care: Systematic review and meta-analysis. J Can Acad Child Adolesc Psychiatry. 2020;29(2):76–105. https://psycnet.apa.org/record/2020-34323-003. 32405310 PMC7213917

[2] Schlack R, Peerenboom N, Neuperdt L, Junker S, Beyer AK. The effects of mental health problems in childhood and adolescence in young adults: Results of the KiGGS cohort. JHealthMonit. 2021;6(4):3–19. 10.25646/8863. 10.25646/8863 PMC8734087 35146318

[3] Sellers R, Warne N, Pickles A, Maughan B, Thapar A, Collishaw S. Cross-cohort change in adolescent outcomes for children with mental health problems. J Child Psychol Psychiatry. 2019;60(7):813–21. 10.1111/jcpp.13029. 30989670 10.1111/jcpp.13029 PMC6617990

[4] UNICEF, Aladro C, Yu R, Sharma M, Ipince A, Bakrania S, Shokraneh F, Sepúlveda J, Anthony D. Mind the gap: child and adolescent mental health and psychosocial support interventions. Florence: UNICEF Office of Research – Innocenti; 2022.

[5] Duarte CS, Lovero KL, Sourander A, Ribeiro WS, Bordin IAS. The child mental health treatment gap in an urban low-income setting: Multisectoral service use and correlates. Psychiatr Serv. 2021;73(1):32–8. 10.1176/appi.ps.202000742. 34106744 10.1176/appi.ps.202000742

[6] Nigg JT. Annual Research Review: On the relations among self-regulation, self-control, executive functioning, effortful control, cognitive control, impulsivity, risk-taking, and inhibition for developmental psychopathology. J Child Psychol Psychiatry. 2017;58(4):361–83. 10.1111/jcpp.12675. 28035675 10.1111/jcpp.12675 PMC5367959

[7] Aldao A, Gee DG, De Los Reyes A, Seager I. Emotion regulation as a transdiagnostic factor in the development of internalizing and externalizing psychopathology: Current and future directions. Dev Psychopathol. 2016;28(4pt1):927–46. 10.1017/S0954579416000638. 27739387 10.1017/S0954579416000638

[8] Morris AS, Criss MM, Silk JS, Houltberg BJ. The impact of parenting on emotion regulation during childhood and adolescence. Child Dev Perspect. 2017;11(4):233–8. 10.1111/cdep.12238.

[9] Havighurst SS, Wilson KR, Harley AE, Prior MR. Tuning in to Kids: An emotion-focused parenting program - Initial findings from a community trial. J Community Psychol. 2009;37(8):1008–23. 10.1002/jcop.20345.

[10] Havighurst SS, Ambrosi CC, Harley A, Kehoe C. Tuning in to Kids: Theoretical basis, program description, and factors impacting effectiveness. J Appl Dev Psychol. 2025;98:101797. 10.1016/j.appdev.2025.10179.

[11] Havighurst SS, Choy R, Ulker A, Otterpohl N, Meybodi FA, Edrissi F, et al. A preliminary evaluation of the cultural appropriateness of the Tuning in to Kids parenting program in Germany, Turkey, Iran and China. Int J Environ Res Public Health. 2022;19(16):10321. https://doi.org/10.3390%2Fijerph191610321. 36011956 10.3390/ijerph191610321 PMC9407904

[12] Zahl-Olsen R, Severinsen L, Stiegler JR, Fernee CR, Simhan I, Rekdal SS. Effects of emotionally oriented parental interventions: A systematic review and meta-analysis. Front Psychol. 2023;14:1159892. 10.3389/fpsyg.2023.1159892. 37519350 10.3389/fpsyg.2023.1159892 PMC10374204

[13] Eurochild. Children’s realities in Europe: progress & gaps. Eurochild 2024 report on children in need across Europe. Brussels: Eurochild; 2024. https://eurochild.org/uploads/2024/11/Eurochild-Flagship-Report-Childrens-Realities-in-Europe.pdf.

[14] Barbabella R, Duarte G, Leão T. The mental health of children and adolescents at Matosinhos: Characteristics and obstacles to care. Eur J Public Health. 2023;33(2):ckad160.771. 10.1093/eurpub/ckad160.1539.

[15] Organisation for Economic Co-operation and Development (OECD). Portugal: perfil de saúde do país 2023 (Portugal: Country Health Profile 2023) (State of Health in the EU). Paris: OECD Publishing; 2023. https://www.oecd.org/pt/publications/portugal-perfil-de-saude-do-pais-2023_6be7d83c-pt.html.

[16] Rainer C, Treloar N, Abdinasir K, Edwards P. A dual crisis: the hidden link between poverty and children’s mental health. London: Centre for Mental Health; 2024.

[17] Serviço Nacional de Saúde (SNS). Tempos médios de resposta para primeiras consultas hospitalares com origem nos cuidados de saúde primários (Average response times for first hospital consultations originating from primary health care). Lisbon: National Health Service; 2025 Sep. https://tempos.min-saude.pt/#/instituicoes-especialidade-cth. Accessed date: January 19, 2026.

[18] Su J. A cross-cultural review of parenting styles: Examining the universality and cultural specificity of authoritative and authoritarian approaches. Lect Note Educ Psychol Public Media. 2025;72:69–74. 10.54254/2753-7048/2025.21297.

[19] Ramos A, Vala J. Are childrearing values’ preferences in Europe associated to socioeconomic development and social inequalities? In: Luijkx R, Reeskens T, Sieben I, editors. Reflections on European values: honouring Loek Halman’s contribution to the European Values Study. Tilburg: Open Press TiU; 2022. pp. 301–19. 10.26116/09eq-y488.

[20] Agbaria Q, Mahamid F. The association between parenting styles, maternal self-efficacy, and social and emotional adjustment among Arab preschool children. Psychol Rev. 2023;36(1):10–23. 10.1186/s41155-023-00252-4. 10.1186/s41155-023-00252-4 PMC10133425 37099037

[21] Nunes C, Bodden D, Lemos I, Lorence B. Parenting practices and quality of life in Dutch and Portuguese adolescents: A cross-cultural study. Rev psicodidáct. 2014;19(2):327–46. 10.1387/RevPsicodidact.10493.

[22] Raudino A, Murray L, Turner C, Tsampala E, Lis A, De Pascalis L, et al. Child anxiety and parenting in England and Italy: The moderating role of maternal warmth. J Child Psychol Psychiatry. 2013;54(12):1318–26. 10.1111/jcpp.12105. 23826833 10.1111/jcpp.12105

[23] Lozada F, Halberstadt AG. Early emotional development and cultural variability. IESBS. 2015;746–51. 10.1016/b978-0-08-097086-8.23007-9.

[24] Choirunnisa I, Ramadhani FE, As S, Safitri S. Overprotective parenting patterns and its influence on children’s mental health. Progres Pendidikan. 2025;6(1):74–81. 10.29303/prospek.v6i1.1225.

[25] Diário da República Eletrónico. Decreto-Lei n.º 59/2007 de 4 de setembro (Decree-Law No. 59/2007 of September 4). Lisbon: National Printing House and Mint. 2007 Sep 4. https://dre.pt. Accessed date: January 19, 2026.

[26] Abrahamyan A, Soares S, Fraga S, Barros H. Prevalence of parental violent discipline toward children: Findings from a Portuguese population. J Interpers Violence. 2024;39(9–10):1881–904. 10.1177/08862605241230552. 38348947 10.1177/08862605241230552 PMC10993632

[27] Tăut D, Băban A, Frantz I, Dănilă I, Lachman JM, Heinrichs N, et al. Prevention of child mental health problems through parenting interventions in Southeastern Europe (RISE): Study protocol for a multi-site randomised controlled trial. Trials. 2021;22(1):960. 10.1186/s13063-021-05817-1. 34961518 10.1186/s13063-021-05817-1 PMC8710933

[28] Kirkbride JB, Anglin DM, Colman I, Dykxhoorn J, Jones PB, Patalay P, et al. The social determinants of mental health and disorder: Evidence, prevention and recommendations. World Psychiatry. 2024;23(1):58–90. 10.1002/wps.21160. 38214615 10.1002/wps.21160 PMC10786006

[29] Lacey RE, Howe LD, Kelly-Irving M, Bartley M, Kelly Y. The clustering of adverse childhood experiences in the Avon longitudinal study of parents and children: Are gender and poverty important? J Interpers Violence. 2020;37(5–6):2218–41. 10.1177/0886260520935096. 32639853 10.1177/0886260520935096 PMC8918866

[30] Lunkenheimer E, Ram N, Skowron EA, Yin P. Harsh parenting, child behavior problems, and the dynamic coupling of parents’ and children’s positive behaviors. J Fam Psychol. 2017;31(6):689–98. 10.1037/fam0000310. 28333490 10.1037/fam0000310 PMC5608615

[31] Negi S, Leerkes EM, Sattler KMP. Indirect pathways from sociodemographic risk to mothers’ supportive emotion socialization via psychological distress and social cognition. J Child Fam Stud. 2025;34:256–69. 10.1007/s10826-024-02969-x.

[32] Dickson SJ, Bussey K, Kangas M, Grocott S, Rapee RM. Barriers to accessing and engaging with mental health services for low-income families in Australia: A qualitative evaluation. J Child Fam Stud. 2025;34:2862–77. 10.1007/s10826-025-03172-2.

[33] Gondek D, Patalay P, Lacey RE. Adverse childhood experiences and multiple mental health outcomes through adulthood: A prospective birth cohort study. SSM Ment Health. 2021;1:100013. 10.1016/j.ssmmh.2021.100013.

[34] Pedersen GA, Smallegange E, Coetzee A, Hartog K, Turner J, Jordans MJD, et al. A systematic review of the evidence for family and parenting interventions in low- and middle-income countries: Child and youth mental health outcomes. J Child Fam Stud. 2019;28:2036–55. 10.1007/s10826-019-01399-4.

[35] Berry V, Melendez-Torres GJ, Axford N, Axberg U, de Castro BO, Gardner F, et al. Does social and economic disadvantage predict lower engagement with parenting interventions? An integrative analysis using individual participant data. Prev Sci. 2022;24(8):1447–58. 10.1007/s11121-022-01404-1. 35870094 10.1007/s11121-022-01404-1 PMC10678811

[36] Pote I, Doubell L, Brims L, Larbie J, Stock L, Lewing B. Engaging disadvantaged and vulnerable parents: an evidence review. London: Early Intervention Foundation; 2019. https://www.eif.org.uk/report/engaging-disadvantaged-and-vulnerable-parents-an-evidence-review.

[37] Pluye P, Tskhay A, Grad R, Barwick M, Doray G, Bartlett-Esquilant G, et al. Low SES parents report more benefits of trustworthy easy-to-read web-based parenting information: A 4-year time series. Ann Fam Med. 2023;21(Suppl1):3628. 10.1370/afm.21.s1.3628.

[38] Nogueira S, Canário AC, Abreu-Lima I, Teixeira P, Cruz O. Group Triple P intervention effects on children and parents: A systematic review and meta-analysis. Int Int J Environ Res Public Health. 2022;19(4):2113. 10.3390/ijerph19042113. 35206299 10.3390/ijerph19042113 PMC8872306

[39] Wandersman A, Imm P, Chinman M, Kaftarian S. Getting to outcomes: A results-based approach to accountability. Eval Program Plann. 2000;23(3):389–95. 10.1016/S0149-7189(00)00028-8.

[40] American Psychological Association. Ethical principles of psychologists and code of conduct. Washington (DC): American Psychological Association; 2010. http://www.apa.org/ethics/code/principles.pdf.

[41] World Medical Association. World Medical Association Declaration of Helsinki: ethical principles for medical research involving human subjects. JAMA. 2013;310(20):2191–4. 10.1001/jama.2013.281053. 24141714 10.1001/jama.2013.281053

[42] Chan A, Tetzlaff JM, Altman DG, Laupacis A, Gøtzsche PC, Krleža-Jerić K, et al. SPIRIT 2013 statement: defining standard protocol items for clinical trials. Annal Intern Med. 2013;158:200–7. 10.7326/0003-4819-158-3-201302050-00583 PMC5114123 23295957

[43] Hopewell S, Chan AW, Collins GS, Hróbjartsson A, Moher D, Schulz KF, et al. CONSORT 2025 statement: updated guideline for reporting randomised trials. BMJ (Clinical Res ed). 2025;389:e081123. 10.1136/bmj-2024-081123. 10.1136/bmj-2024-081123 PMC11995449 40228833

[44] Mohr DC, Spring B, Freedland KE, Beckner V, Arean P, Hollon SD, et al. The selection and design of control conditions for randomized controlled trials of psychological interventions. Psychother Psychosom. 2009;78(5):275–84. 10.1159/000228248. 19602916 10.1159/000228248

[45] Faria MR, Alves S. Exploring parental acceptability and preferences for emotion-focused parenting programs [Manuscript submitted for publication]. School of Psychology and Life Sciences, Lusófona University; 2025.

[46] Meybodi FA, Mohammadkhani P, Pourshahbaz A, Dolatshahi B, Havighurst SS. Improving parent emotion socialization practices: Piloting Tuning in to Kids in Iran for children with disruptive behavior problems. Fam Relat. 2019;68(5):596–607. 10.1111/fare.12387.

[47] Qiu C, Shum KK. Emotion coaching intervention for Chinese mothers of preschoolers: A randomized controlled trial. Child Psychiatry Hum Dev. 2022;53:61–75. 10.1007/s10578-020-01101-6. 33389390 10.1007/s10578-020-01101-6

[48] Bølstad E, Havighurst SS, Tamnes CK, Nygaard E, Bjørk RF, Stavrinou M. A pilot study of a parent emotion socialization intervention: Impact on parent behavior, child self-regulation, and adjustment. Front Psychol. 2021;12:730278. 10.3389/fpsyg.2021.730278. 34721193 10.3389/fpsyg.2021.730278 PMC8554311

[49] Eisenberg N, Cumberland A, Spinrad TL. Parental socialization of emotion. Psychol Inq. 1998;9(4):241–73. 10.1207/s15327965pli0904_1. 16865170 10.1207/s15327965pli0904_1 PMC1513625

[50] Gottman JM, Katz LF, Hooven C. Meta-emotion: how families communicate emotionally. Mahwah (NJ): Lawrence Erlbaum Associates; 1997.

[51] Havighurst SS, Radovini A, Hao B, Kehoe CE. Emotion-focused parenting interventions for prevention and treatment of child and adolescent mental health problems: A review of recent literature. Curr Opin Psychiatry. 2020;33(6):586–601. 10.1097/yco.0000000000000647. 32858599 10.1097/YCO.0000000000000647

[52] Havighurst SS, Harley A. Tuning in to Kids^®^ program manual. 4nd ed. Melbourne: Mindful, The Centre for Training and Research in Developmental Health, The University of Melbourne; 2024.

[53] Fabes RA, Eisenberg N, Bernzweig J. Coping with children’s negative emotions scale (CCNES): description and scoring. Tempe (AZ): Arizona State University; 1990.

[54] Alves D, Cruz O. Reações parentais às emoções negativas dos filhos (RPEM): Um questionário de avaliação da meta-emoção parental (Parental Reactions to Children’s Negative Emotions (CCNES): A questionnaire for assessing parental meta-emotion.) In Actas do VIII Congresso Iberoamericano de Avaliação/Evaluación Psicológica e XV Conferência Internacional Avaliação Psicológica: Formas e Contextos. 2011. https://hdl.handle.net/10216/57244. Accessed date: January 19, 2026.

[55] Pereira AI, Santos C, Barros L, Roberto MS, Rato J, Prata A, et al. Patterns of parental reactions to their children’s negative emotions: A cluster analysis with a clinical sample. Int J Environ Res Public Health. 2022;19:6844. 10.3390/ijerph19116844. 35682427 10.3390/ijerph19116844 PMC9180051

[56] Mirabile SP. Ignoring children’s emotions: A novel ignoring subscale for the Coping with Children’s Negative Emotions Scale. Eur J Dev Psychol. 2015;12(4):459–71. 10.1080/17405629.2015.1037735.

[57] Halberstadt AG, Dunsmore JC, Bryant A, Parker AE, Beale KS, Thompson JA. Development and validation of the Parents’ Beliefs About Children’s Emotions Questionnaire. Psychol Assess. 2013;25(4):1195–210. 10.1037/a0033695. 23914957 10.1037/a0033695 PMC4018216

[58] Caiado B, Moreira H, Canavarro MC. Parents’ beliefs about children’s emotions questionnaire: Psychometric properties of the Portuguese version among a sample of parents of school-aged children. Curr Psychol. 2021;42:16098–110. 10.1007/s12144-021-01504-1.

[59] Duncan L, Coatsworth JD, Greenberg M. A model of mindful parenting: Implications for parent-child relationships and prevention research. Clin Child Fam Psychol Rev. 2009;12(3):255–70. 10.1007/s10567-009-0046-3. 19412664 10.1007/s10567-009-0046-3 PMC2730447

[60] Moreira H, Canavarro MC. Psychometric properties of the Interpersonal Mindfulness in Parenting Scale in a sample of Portuguese mothers. Mindfulness. 2017;8(3):691–706. https://doi.org/10.1007/s12671-016-0647-0. http://link.springer.com/article/.

[61] Kaufman EA, Xia M, Fosco G, Yaptangco M, Skidmore CR, Crowell SE. The Difficulties in Emotion Regulation Scale Short Form (DERS-SF): Validation and replication in adolescent and adult samples. J Psychopathol Behav Assess. 2016;38:443–55. 10.1007/s10862-015-9529-3. 41522882 10.1007/s10862-015-9529-3 PMC12788809

[62] Moreira H, Gouveia MJ, Canavarro MC. A bifactor analysis of the Difficulties in Emotion Regulation Scale - Short Form (DERS-SF) in a sample of adolescents and adults. Curr Psychol. 2020;41:757–82. 10.1007/s12144-019-00602-5.

[63] Goodman R. The Strengths and Difficulties Questionnaire: A research note. J Child Psychol Psychiatry. 1997;38(5):581–6. 10.1111/j.1469-7610.1997.tb01545.x. 9255702 10.1111/j.1469-7610.1997.tb01545.x

[64] Fleitlich B, Loureiro M, Fonseca A, Gaspar MF. Questionário de capacidades e de dificuldades (SDQ-Por) (Strengths and Difficulties Questionnaire – Portuguese version). Portefólio de Modelos e Processos de Avaliação Psicológica; 2005. https://portefolio-mpap.webnode.pt/escala-sdq/. Accessed date: January 19, 2026.

[65] Costa PA, Tasker F, Ramos C, Leal I. Psychometric properties of the parent’s versions of the SDQ and the PANAS-X in a community sample of Portuguese parents. Clin Child Psychol Psychiatry. 2020;25(2):520–32. 10.1177/1359104519891759. 31793797 10.1177/1359104519891759

[66] Shields A, Chichetti D. Emotion regulation among school-age children: The development and validation of a new criterion Q-sort scale. Dev Psychol. 1997;33(6):906–16. 10.1037/0012-1649.33.6.906. 9383613 10.1037//0012-1649.33.6.906

[67] Melo AIMT. Emotions during childhood: parental coping with children’s emotions and children’s externalizing and internalizing symptoms [Master’s dissertation] Braga: University of Minho; 2005. https://hdl.handle.net/1822/4926. Accessed date: January 19, 2026.

[68] Fernandes M, Morais I, Santos C, Guedes M, Ribeiro O, Fernandes C, et al. Factorial structure, measurement invariance and reliability of the Emotion Regulation Checklist (ERC) in a sample of Portuguese parents. Análise Psicológica. 2024;42(2):197–207. 10.14417/ap.2032.

[69] Berry JO, Jones WH. The Parental Stress Scale: Initial psychometric evidence. J Soc Pers Relatsh. 1995;12(3):463–72. 10.1177/0265407595123009.

[70] Mixão M, Leal I, Maroco J. Escala de Stress Parental. In: Leal I, editor. Avaliação em sexualidade e parentalidade. Porto: Livpsic; 2007. pp. 199–210.

[71] Algarvio S, Leal I, Maroco J. (2018). Parental Stress Scale: Validation study with a Portuguese population of parents of children from 3 to 10 years old. J Child Health Care. 2018;22(4):563–576. 10.1177/1367493518764337. 10.1177/1367493518764337 29540078

[72] Zigmond AS, Snaith RP. The Hospital Anxiety and Depression Scale. Acta Psychiatr Scand. 1983;67:361–70. 10.1111/j.1600-0447.1983.tb09716.x. 6880820 10.1111/j.1600-0447.1983.tb09716.x

[73] Pais-Ribeiro, Silva I, Ferreira T, Martins A, Meneses R, Baltar M. Validation study of a Portuguese version of the Hospital Anxiety and Depression Scale. Psychol Health Med. 2007;12(2):225–35. 10.1080/13548500500524088. 17365902 10.1080/13548500500524088

[74] Mourão D, Fonseca A, Moreira H. Psychopathology and mindful parenting in parents of preschool and school-aged children: The role of supportive coparenting. Int J Environ Res Public Health. 2023;20(2):1238. 10.3390/ijerph20021238. 36673983 10.3390/ijerph20021238 PMC9858786

[75] Hamilton VE, Matthews JM, Crawford SB. Development and preliminary validation of a parenting self-regulation scale: Me as a Parent. J Child Fam Stud. 2015;24(10):2853–64. 10.1007/s10826-014-0089-z.

[76] Marques T, Cadete C, Barros L, Pereira AI. Escala de Autorregulação Parental (Parental Self-Regulation Scale) [Unpublished manuscript]. Lisbon, Faculty of Psychology, University of Lisbon; 2015.

[77] Marques T, Pereira AI, Barros L, Roberto MS. Factor structure of the Me as A Parent scale in a community sample of Portuguese mothers. PSICOLOGIA. 2020;34(2):205–14. 10.17575/psicologia.v34i2.1637.

[78] Braun V, Clarke V. Conceptual and design thinking for thematic analysis. Qual Psychol. 2022;9(1):3–26. 10.1037/qup0000196.

[79] Lorah J. Effect size measures for multilevel models: Definition, interpretation, and TIMSS example. Large scale Assess Educ. 2018;6:8. 10.1186/s40536-018-0061-2.

